# Bacterial Leakage of Mineral Trioxide Aggregate, Calcium-Enriched Mixture and Biodentine as Furcation Perforation Repair Materials in Primary Molars

**DOI:** 10.7508/iej.2016.03.013

**Published:** 2016-05-01

**Authors:** Nahid Ramazani, Parisa Sadeghi

**Affiliations:** a *Children and Adolescent Health Research Center, Oral and Dental Disease Research Center, Department of Pediatric Dentistry, School of Dentistry, Zahedan University of Medical Sciences, Zahedan, Iran; *; b * Dentist, Zahedan University of Medical Sciences, Zahedan, Iran*

**Keywords:** Biodentine, Biomaterial, Calcium-Enriched Mixture, Furcation Perforation, Mineral Trioxide Aggregate, Perforation Repair, Sealability

## Abstract

**Introduction:**

Adequate seal of iatrogenically perforated area within the root canal system can improve the long term treatment prognosis. This *in vitro* study evaluated the sealing ability of mineral trioxide aggregate (MTA), calcium-enriched mixture (CEM) cement and Biodentine in repair of furcation perforation in primary molars.

**Methods and Materials:**

A total of 61 freshly extracted primary mandibular second molars were randomly divided into three groups (*n*=17) and 10 teeth were put in negative (without perforation, *n*=5) and positive (perforated without repair, *n*=5) control groups. Turbidity was used as the criteria of bacterial leakage, when detected in the model of dual-chamber leakage. Data were analyzed using the Chi-Square and Kaplan-Meier survival analysis in SPSS software. The level of significance was set at 0.05.

**Results:**

All positive samples showed turbidity, whereas none of the negative samples allowed bacterial leakage. There was no significant difference between the number of turbidity samples in repaired teeth with all test materials (*P*=0.13). No significant difference was also detected in the mean survival time (*P*>0.05).

**Conclusion:**

CEM cement and Biodentine showed promising results as perforation repair materials and can be recommended as suitable alternatives of MTA for repair of furcation perforation of primary molars.

## Introduction

Dental caries is the most prevalent infectious disease during childhood [1]; most of which necessitate pulp treatment. The main objective of pulp therapy in primary teeth is to maintain the integrity of oral structures, guide permanent teeth to erupt properly and finally ensure general well-being of the child [2]. Because of the complexity of treatments and behavior challenges in children [3], procedural accidents such as perforation and creating an artificial opening in the furcation area are not uncommon. Perforation has been reported to occur in 2-12% of cases [4]. This procedural accident influences the prognosis of endodontic treatment [5, 6]. 

Repair of the perforation with an optimum sealing biomaterial has clinical significance in preventing the consequences and eventual tooth loss [6, 7]. In recent years, the sealing ability of the perforation repair material has been the subject of many investigations. Although it cannot be considered as the only criterion, providing adequate seal at the interface of material-dentin wall is one of the characteristics of ideal repair material [7]. Different biomaterials show different degree of sealing ability and biocements like mineral trioxide aggregate (MTA) and calcium-enriched mixture (CEM) cement have been successfully used for this purpose [5, 6, 8].

MTA has resulted in successful outcomes in furcation repair. Despite many good properties [9], MTA has long setting time and difficult handling [5, 10-12]. These are important considerations for clinical application in pediatric patients. CEM is cement also provides good results when used for perforation repair [5, 10]. This cement has short setting time and offers good sealing ability and handling [5, 8, 10, 13, 14]. Biodentine is another popular biomaterial in endodontics with promising results. It is a new dentine substitute containing tricalcium silicate with good handling and mechanical properties. It has also good sealing ability and short setting time [4, 11, 12, 15-19]. In two separate studies, Haghgoo *et al.* [5, 8], found no significant difference between MTA and CEM cement as perforation repair materials in primary molars. There are also sparse papers that have evaluated the applications of Biodentine as a posterior restorative material [15], the capping agent in vital pulp therapies [18, 20, 21] and root end filling [17].

Given the serious implications of furcation perforation as well as sparse data comparing the sealing ability of MTA, CEM cement and Biodentine for repairing the perforated primary teeth, the purpose of this *in vitro* study was to compare the bacterial leakage of MTA, CEM cement and Biodentine in repairing the simulated furcation perforations in primary molars using dual-chamber bacterial leakage model.

## Materials and Methods

Ethics Committee of Zahedan University of Medical Sciences approved the study protocol (Grant No: 7121). In this *in vitro* study, 61 freshly extracted primary mandibular second molars were used. The sample size was calculated based on previous similar studies [8, 22, 23] using Minitab statistical software. The inclusion criteria were as follows: normal furcation (with completely distinct roots), minimal caries (at least 4 mm caries free surfaces above the CEJ), and no previous pulp treatment. Teeth with cracks were excluded after microscopic inspection. After cleaning, washing and disinfecting, the samples were kept in normal saline (0.9% NaCl, Darupakhsh, Tehran, Iran) until used. The samples were then horizontally sectioned 2 and 4 mm away from CEJ apically and occlusally, respectively using a high speed diamond disk, (Dorsa, HLF 86, Tehran, Iran) with water cooling.

In each tooth, the access cavity was prepared using a #5 diamond bur (D&Z Co., Wies Baden, Germany) mounted in a high speed water cooled handpiece. Cavity preparations with 2 mm depths were also made at root ends. Then, orifices and the prepared apical-end cavities of roots were filled with light-cured glass ionomer (Fuji II LC, GC Corporation, Tokyo, Japan). Except for the negative control group, the floor of pulp chamber was perforated using a #010 round bur (D&Z Co., Wies Baden, Germany) installed on a high speed handpiece with constant water spray. The size of perforation was the same as the bur size (1 mm in diameter) in all samples. The bur was replaced with a new one after making every six perforations.

The samples were randomly assigned into five groups (three experimental and two control groups). In groups I, II and III (*n*=17), perforations were sealed with either ProRoot MTA (Dentsply, Tulsa Dental, Tulsa, OK, USA), CEM cement (BioniqueDent, Tehran, Iran) or Biodentine (Septodont, Saint-Maur-des-Fosses, France), respectively. In the positive control group (*n*=5), no repairing material was used. In the negative control group (*n*=5), the furcation area was covered with two coats of nail varnish. After irrigation of samples with 10 mL normal saline, repairing materials were mixed according to the manufactures’ instructions and placed by a carrier gun on the perforation site. Biomaterials were packed with moist cotton pellets while the samples were positioned in wet soft sponges. Condensing upon the sponge simulated the clinical condition in the oral cavity. At the end, all samples were placed in an incubator at 37^°^C and 100% humidity for 24 h to allow the biomaterials being fully set. Subsequently, teeth were coated by two layers of nail varnish except for the perforation site and nearly 1 mm around it. 

In this experiment, a dual-chamber anaerobic bacterial leakage apparatus was used. The upper chamber was assembled by 3 mL plastic Eppendorf cylinder (Sigma-Aldrich Co., Hamburg, Germany) after cutting off 5 mm from its end. The samples were mounted in the cylinder so that the external surface of perforation area was left outside and accessible. The gaps between the sample and the inner side of cylinder were completely sealed with sticky wax. The apparatus was sterilized with ethylene oxide for 8 h. The upper chamber was inserted in 10 mL glass vial (Pouyan Teb Co., Tehran, Iran) as the lower chamber which previously was filled with 5 mL of sterile Phenol Red Broth (PRB, Merck, Darmstadt, Germany). The upper-lower chamber interface was tightly sealed with parafilm (Supa Co., Tehran, Iran). It was checked that the perforation area was immersed in PRB. The whole assembling was incubated at 37^°^C in 100% humidity for 3 days. An amount of 9×10^8 ^CFU/mL of *Enterococcus faecalis* (*E. faecalis*) (PTCC1778) compatible with 0.5 McFarland standard was used to inoculate 2 mm of PRB. This bacterial suspension was added to the upper chamber every two days. The vial glasses were daily observed for turbidity (red to yellow color conversion) as the indicator of bacterial growth throughout 90 days of experiment. 

In this study, all procedures were done by the same experienced practitioner. Data were recorded for each experimental or control samples and finally analyzed using the Chi-Square and Kaplan-Meier survival analysis tests with SPSS software (SPSS version 18.0, SPSS, Chicago, IL, USA). The level of significance was set at 0.05.

## Results

During the entire observational period, control samples behaved as were expected. All positive samples exhibited red to yellow color changes. Whiles, no color conversion was recorded with the negative control group. 

The majority of MTA samples remained without turbidity throughout the monitoring period. On the 34^th^ day of apparatus assembling, color conversion of one sample in MTA group was detected. There was another sample with color change recorded by day 48 in this group. 

Turbidity did not occur until day 27^th^ in CEM samples. During the experimental period, three additional samples from CEM group exhibited bacterial contamination on day 40. Two of the remaining samples showed turbidity on day 49^th^. The first two samples of Biodentine group showed leakage observed by day 23. The other turbidity samples were added by day 25 (one sample), day 48 (two samples) and day 62 (two samples).

Throughout the experiment, the turbidity results remained unchanged from day 62 to the end. Totally, as presented in Table 1, MTA showed the less number of turbidity followed by CEM cement and Biodentine, respectively. However, the Chi-Square test failed to detect a statistical significant difference among three experimental groups (*P*=0.13). The means of survival time in different groups are shown in Table 2. According to the Kaplan-Meier survival analysis, there was no significant difference in mean survival time of study groups (*P*>0.05).

## Discussion

The focus point of the current study (comparison of MTA, CEM cement and Biodentine based on the sealing ability as perforation repair materials) have not been explored in earlier studies. Totally, 2, 6 and 7 samples of turbidity were recorded with MTA, CEM cement and Biodentine, respectively. Although, based on statistical tests, throughout the 90-day experiment, perforation repair material had no significant effect on the microbial leakage.

Different techniques have been proposed to assess the sealing ability of various perforation repair materials [9, 24, 25]. Microleakage dye penetration model is one of the traditional methods with advantages such as easy manipulation and inexpensiveness [16, 26]. However, chemical characteristics, pH and low molecular size of dye and it’s dissolution by repairing material may affect the depth of dye penetration and cause leakage to be over- or under-estimated [8, 13, 26]. Moreover, it has to be noted that no more than one plan of dye penetration can be detected [26]. These make the above mentioned method not clinically relevant and it is logical to adopt a new standard, valid and reliable model. So in the current study, microleakage analysis method using a dual-chamber microleakage apparatus utilizing *E. faecalis, *was applied*.* This method is an improvement over microleakage dye penetration model. By using this assembling, the clinical bacterial contamination can be simulated [13, 27]. *E. faecalis* was chosen because it is the commonly detected microorganism in post endodontic failure [27]. This gram positive, facultative, anaerobic organism has the ability to persist with inadequate source of nutrition and can invade the dentinal tubules [5, 9, 28, 29].

Several studies have reported the ability of MTA to prevent leakage in a variety of applications [8, 14, 20, 24, 30]. Moreover, they reported its superiority compared to other dental materials. For this reason, we included MTA as a standard perforation repair material for better comparison. This material is routinely used to repair peroration defects mainly due to its moist compatibility [31-33]. In the study by Sahebi *et al*. [6], regarding the sealing ability of different materials, significantly more microleakage of MTA was reported compared to CEM cement. However, the present research found no detectable difference between MTA and CEM cement in this regard. The differences in methodology can be the reason that makes direct comparison difficult. Perhaps the techniques used to evaluate microleakage and the type of tooth material (primary versus permanent teeth) may be related to the different findings. 

In another *in vitro* study CEM cement exhibited no significant difference from MTA as root-end filling and sealing materials [25]. Although in that study a method other than dual-chamber apparatus was used. However, in one recent study by Zarenejad *et al.* [14], CEM cement and MTA showed similar behaviors when applied as intra-orifice barrier during nonvital bleaching.

**Table 1:** Turbidity in different experimental groups and the time of detection and number of samples added

**Group**	**Turbidity**	**Day (N)**
**MTA**	2	34 (1) 48 (1)
**CEM cement**	6	27 (1) 40 (3) 49 (2)
**Biodentine**	7	23 (2) 25 (1) 48 (2) 62 (2)

**Table 2:** Mean (SD) of survival time in different experimental groups [Confidence Interval (CI) =95%]

**Group**	**Mean (SD)**
**MTA**	84.235 (3.873)
**CEM cement**	72.647 (5.797)
**Biodentine**	70.059 (6.319)
**Total**	75.647 (3.253)

The findings of the current study confirm those of two previous investigations by Haghgoo *et al.* [5, 8] who evaluated the sealing ability of CEM cement and MTA by using bacterial leakage and dye penetration models in primary teeth. They concluded that the two tested biomaterials demonstrate similar capacities as furcation perforation materials [5, 8]. 

The results of the study by Shahi *et al.* [26], using protein leakage model in permanent teeth, confirms the findings of the present study regarding the good sealing ability of MTA as perforation repair material. In addition, in another study by Samiee *et al.* [34], MTA and CEM allowed similar resistance to leakage at the material-dentinal wall interface of repaired perforations. The results obtained from that study is consistent with the result yielded in the present investigation. CEM cement when compared to MTA as driven from different studies [13, 35, 36], and the current one revealed no detectable difference or even statistically superior performance. Considering the result obtained together with the benefits such as short setting time, low toxicity and low price [5, 27, 29, 37] CEM cement can be proposed as potential substitute of MTA. 

Different applications have been proposed for Biodentine [15, 17, 18, 20, 21, 38]. Some studies investigated the performance of this new dentine substitute in restoration of posterior teeth [15, 16]. Koubi *et al.* [16], used glucose diffusion microleakage method and found that the material performed as well as resin modified glass ionomer. Additionally, less marginal discoloration and good handling has been attributed to Biodentine [15]. In one study, it was showed that when Biodentine was used as root-end filling material, significantly better marginal adaptation was observed compared to MTA [17]. However, according to Soundappan *et al.* [12], Biodentine could not compete with MTA as root end filling material. Considering the biocompatible entity of Biodentine and its ability to induce odontoblast differentiation the bacterial leakage resistance of this calcium-silicate cement after repair of perforation must be assessed. Given the good properties of Biodentine [11, 20] together with our findings addition of this cement to the list of primary tooth perforation repair materials is crucial. 

MTA, CEM cement and Biodentine participate in hydroxyl apatite formation at the material-dentine interface [14, 39]. After adding liquid to powder, these three formulations form small sized non structured hydrate gels, which may flow to better accessing gaps and spread and fit into the dentinal tubules by wetting of dentin surface which in turn prevents bacterial leakage. Moreover, they exhibited a slight post-setting expansion [5, 12, 15]. The amount of bacterial leakage is proportional to the size of perforation [16]. In order to achieve a valuable comparison, all perforation defects were made similarly and with the same size. 

Despite the promising results regarding the sealing ability of CEM cement and Biodentine, it should be kept in mind that *in vitro* studies, due to many inherent drawbacks cannot simulate oral condition completely. On the other hand, because of no expression of full clinical characteristic of the repairing material under *in vitro* conditions, the long term prognosis of perforation sealed teeth are unknown. So future clinical studies on accidentally perforated primary molars are recommended to evaluate the long term prognosis.

## Conclusion

Within the limitation of the present study, CEM cement and Biodentine had no notable difference compared to MTA in terms of *in vitro* bacterial leakage. 
